# Comparison of the NIST and BIPM Air-Kerma Standards for Measurements in the Low-Energy X-Ray Range

**DOI:** 10.6028/jres.104.009

**Published:** 1999-04-01

**Authors:** D. T. Burns, P. Lamperti, M. O’Brien

**Affiliations:** Bureau International des Poids et Mesures, F-92312 Sèvres Cedex France; National Institute of Standards and Technology, Gaithersburg, MD 20899-0001, USA

**Keywords:** air kerma, free-air ionization chamber, primary standard, reference radiation qualities

## Abstract

A direct comparison was made between the air-kerma standards used for the measurement of low-energy x rays at the National Institute of Standards and Technology (NIST) and the Bureau International des Poids et Mesures (BIPM). The comparison was carried out at the BIPM using the BIPM reference beam qualities in the range from 10 kV to 100 kV. The results show the standards to be in agreement to around 0.5 % at reference beam qualities up to 50 kV and at 100 kV. The result at the 80 kV beam quality is less favorable, with agreement at the 1 % level.

## 1. Introduction

A direct comparison was made between the air-kerma primary standards for low-energy x rays of the National Institute of Standards and Technology (NIST) and the Bureau International des Poids et Mesures (BIPM). The comparison took place at the BIPM in July 1998 using the BIPM tungsten reference radiation qualities in the range from 10 kV to 100 kV, which are the reference conditions recommended by the Consultative Committee for Ionizing Radiation (CCEMRI) [1]. Two NIST primary-standard free-air ionization chambers were transported to the BIPM for the comparison. The Lamperti chamber operates in the range from 10 kV to 60 kV and is the U.S. national standard for beam qualities of 15 kV and below. The Ritz chamber operates in the range from 20 kV to 100 kV and is the U.S. national standard for beam qualities in this range. The BIPM standard is designed for use in the range from 10 kV to 100 kV.

### 2. Determination of the Air-Kerma Rate

For a free-air ionization chamber standard with measuring volume V, the air-kerma rate is determined by the relation

$$\dot{K}=\frac{I}{\rho_{\text{air}}V}\frac{W_{\text{air}}}{e}\frac{1}{1-g_{\text{air}}}\prod_{i}k_{i},$$ (1)

where

*I*/*ρ*_air_*V* is the mass ionization current measured by the standard,

*W*_air_ is the mean energy expended by an electron of charge *e* to produce an ion pair in dry air,

*g*_air_ is the fraction of the initial electron energy lost by bremsstrahlung production in air, and

*∏ k_i_* is the product of the correction factors to be applied to the standard.

The values for the physical constants used in the determination of the air-kerma rate are given in Table 1.

## 3. Details of the Standards

All three free-air chamber standards used in the present comparison are of the conventional parallel-plate design; the measuring volume *V* is defined by the diameter of the chamber aperture and the length of the collecting plate. The BIPM air-kerma standard is described in Refs. [2] and [3]. The NIST Lamperti standard was previously compared with the BIPM standard in a direct comparison carried out at the BIPM in 1966, the results of which are reported in Ref. [2] and which also gives details of the Lamperti chamber, as does Ref. [4]; the chamber employs a guard-ring system to establish a uniform electric field. Details concerning the Ritz standard, which has not previously been compared with the BIPM standard, can be found in Ref. [5]; this chamber uses a guard plate and guard-strip system to minimize distortion of the electric field. The main dimensions, the measuring volume, and the polarizing voltage for all three chambers are given in Table 2.

## 4. Comparison Details

### 4.1 BIPM Irradiation Facility and Reference Beam Qualities

The comparison was carried out in the BIPM low-energy x-ray laboratory, which houses a constant-potential generator (maximum usable generating potential 100 kV) and a tungsten-anode x-ray tube with an inherent filtration of 2.9 mm beryllium. Both the generating potential and the tube current are stabilized using feedback systems constructed at the BIPM; this results in very high stability and obviates the need for a transmission current monitor. The variation in the measured ionization current over the duration of a comparison is typically 0.04 %. The radiation qualities used in the range from 10 kV to 50 kV are those recommended by CCEMRI [1] and are given in Table 3 (in ascending half-value layer from top to bottom). For the Ritz standard, measurements were also made at the 80 kV and 100 kV qualities given in the table.

### 4.2 Correction Factors

Although free-air chambers are designed to minimize or eliminate corrections to the measured ionization current, certain corrections are unavoidable. The correction factors applied to each chamber at each radiation quality, together with their associated uncertainties, are given in Tables 4 to 6.

The largest correction at low energies is that due to the attenuation of the x-ray fluence along the air path of length *L* between the reference plane and the center of the collecting volume. The correction factor *k*_a_ is calculated using the measured air attenuation coefficients *μ*_air_ given in Table 3 (in units of inverse length), according to

$$k_{\text{a}}=\exp(\mu_{\text{air}}L)$$ (2)

In practice, the values used deviate slightly from those given in the tables. This is because the attenuation varies with the temperature *T* and pressure *p* of the air in the chamber and the values for *k*_a_ used are corrected for this effect. The value for *k*_a_ for the Lamperti chamber at 10 kV has been increased by the factor 1.0009 to account for the larger mean air attenuation coefficient for an air path of 39.2 mm (the values given in Table 3 were measured at the BIPM for an air path of 100 mm). This effect is negligible at the other beam qualities. All ionization measurements are also corrected for the temperature and pressure of the ambient air between the radiation source and the reference plane using the air attenuation coefficients given in Table 3.

All measured ionization currents are corrected for ion recombination. For the NIST chambers, the values for *k*_s_ given in Tables 4 and 5 are derived from measurements at NIST using the BIPM air-kerma rates given in Table 3. For comparison with the Ritz standard, the usual BIPM aperture of diameter 4.9992 mm was replaced by an aperture of diameter 9.941 mm; values for *k*_s_ for each BIPM aperture diameter are given in Table 6.

### 4.3 Chamber Positioning and Measurement Procedure

For each comparison, the appropriate NIST chamber was positioned close to the BIPM chamber and both chambers remained fixed in position throughout a comparison; alternation between chambers was carried out by displacement of the radiation source. Alignment on the beam axis was measured to around 0.1 mm and this position was reproducible to better than 0.01 mm. A movement of 0.1 mm off-axis was shown to change the measured current by around 0.03 % at 10 kV and at 50 kV. No correction is applied for the radial nonuniformity of the beam since the aperture diameters are closely matched. The reference plane for each chamber was positioned at 500 mm from the radiation source for all qualities up to 50 kV and at 750 mm for the 80 kV and 100 kV qualities. This distance was measured to 0.03 mm and was reproducible to better than 0.01 mm. The beam diameter in the reference plane is 45 mm for all qualities up to 50 kV, 68 mm for the 80 kV quality, and 135 mm for the 100 kV quality.

The leakage current was measured before and after each series of ionization current measurements and a correction made based on the mean of these leakage measurements. For the Lamperti chamber the leakage current was less than 0.02 % of the ionization current and for the Ritz and BIPM chambers less than 0.03 %. Each measurement series consisted of five measurements, the integration time for each measurement being between 20 s and 80 s. The relative standard uncertainty of the mean of a series of five measurements was typically less than 0.02 %, in the worst case 0.04 %. Taking into account a typical variation in the measured ionization current of 0.04 % over the duration of a comparison, a Type A standard uncertainty of 0.05 % is taken for all current measurements. For all chambers, measurements were made at both polarities to correct for any polarity effect. The measured difference was typically 0.11 % for the BIPM chamber, 0.02 % for the Lamperti chamber, and around 0.06 % for the Ritz chamber.

Due to a failure of the NIST temperature probe, the air temperature for the Lamperti chamber was taken to be that measured by the thermistor positioned in the BIPM capacitor bank. A series of tests showed this procedure to be reliable to better than 0.05 K. For the Ritz chamber, the air temperature was measured using a BIPM mercury thermometer calibrated to 0.02 K and positioned in the holder of the Ritz chamber. For the Ritz chamber the polarizing voltage was applied for the duration of the comparison so as to maintain temperature equilibrium. All ionization measurements are corrected to a temperature of *T* = 273.15 K and a pressure of *p* = 101325 Pa.

## 5. Measurement Uncertainties

The uncertainties associated with the primary standards and with the results of the comparison are given in Table 7. The NIST uncertainties were evaluated according to Ref. [6]. In general, the quoted uncertainties in the air-kerma rate determination are representative of those associated with routine air-kerma rate determinations at both institutions. The uncertainties associated with the measurement of the ionization current and with chamber positioning are those which apply to measurements at the BIPM and are different from those in routine use for air-kerma rate determinations at NIST.

The uncertainties of the ratios ${\dot{K}}_{\text{NIST}}/{\dot{K}}_{\text{BIPM}}$ take into account correlations due to common Type B uncertainties associated with the measurement of the ionization current, the humidity correction and the physical constants. Correlations between the values for *k*_sc_ are not taken into account, although these are derived from the same data set.

## 6. Results and Discussion

The results for the Lamperti standard are shown in Table 8. The Lamperti and BIPM standards agree at around the 0.3 % to 0.6 % level, which is one to two times the standard uncertainty. The results are slightly further from unity than those of the previous comparison in 1966, particularly at the lower beam qualities. Small changes in the values adopted for *k*_sc_ and *k*_s_ for both standards account for around 0.05 % of the difference between the new and old results.

The results for the Ritz standard are given in Table 9. Agreement with the BIPM standard at the 0.5 % level is observed, except for the 80 kV beam quality. This result at 80 kV is likely to be due to the value used for the electron-loss correction *k*_e_ for the Ritz standard at this quality. The electron-loss and photon-scatter correction factors for the Ritz chamber are currently being re-evaluated for some new reference radiation qualities under development at NIST and a change to the value for *k*_e_ at 80 kV is expected. The present comparison results are based on the values for the correction factors available at the time of the comparison. In addition, Monte Carlo calculations of *k*_e_ and *k*_sc_ are in progress at the BIPM [7], including calculations for the Lamperti and Ritz chambers.

## Figures and Tables

**Table 1:** Physical constants used in the determination of the air-kerma rate

Physical constant	Value	Relative standard uncertainty (%)
*ρ*_air_^a^	1.293 kg m^−3^	0.01
*W*_air_/*e*	33.97 J C^−1^	0.15
1 − *g*_air_	1.000 0	0.01

a Density of dry air at *T* = 273.15 K and *p* = 101 325 Pa.

**Table 2:** Main characteristics of the primary standards used in the comparison

Characteristic	NIST Lamperti	NIST Ritz	BIPM
Air-path length (mm)	39.2	127.4	100.0
Plate separation (mm)	40	90	70
Collecting plate length (mm)	10.135	70.03	15.466
Aperture diameter (mm)	4.994 3	10.001 7	4.999 2^a^
Measuring volume (mm^3^)	198.55	5 502.0	303.58^a^
Polarizing voltage (V)	1 500	5 000	1 500

a For comparison with the Ritz standard an aperture of diameter 9.941 mm was used in the BIPM standard, which gives a measuring volume of 1200.4 mm^3^.

**Table 3:** Characteristics of the BIPM reference radiation qualities used for the comparison

Generating potential (kV)	Aluminium filtration (mm)	Aluminium half-value layer (mm)	Air attenuation coefficient^a^ (10^−3^ mm^−1^)	Air-kerma rate (mGy s^−1^)
10	0	0.036	1.757	0.56
30	0.2082	0.176	0.415	3.31
25	0.3734	0.250	0.304	1.13
50(b)	1.0082	1.021	0.091	1.57
50(a)	3.989	2.257	0.046	0.34
80	3.041	3.01	0.042	0.61
100	3.451	4.00	0.035	0.84

a At *T* = 293.15 K and *p* = 100 000 Pa, measured at the BIPM for an air-path length of 100 mm.

**Table 4:** Correction factors used in the comparison for the Lamperti standard

Correction factor	Generating potential (kV)					Relative standard uncertainty (%)	
	10	30	25	50(b)	50(a)	Type A	Type B
Air attenuation *k*_a_^a^	1.072 1	1.016 4	1.012 0	1.003 6	1.001 8	0.03	0.01
Scattered radiation *k*_sc_	0.996 0	0.996 7	0.996 9	0.997 5	0.997 9		0.20
Electron loss *k*_e_	1.000	1.000	1.000	1.001	1.005		0.10
Ion recombination *k*_s_	1.000 0	1.000 1	1.000 0	1.000 0	1.000 0	0.04	
Field distortion *k*_d_	1.000	1.000	1.000	1.000	1.000		0.20
Aperture transmission *k*_1_^b^	1.000 0	1.000 0	1.000 0	1.000 0	1.000 2		
Wall transmission *k*_p_^b^	1.000 0	1.000 0	1.000 0	1.000 0	1.000 0		
Humidity *k*_h_	0.998	0.998	0.998	0.998	0.998		0.10

a These are nominal values for *T* = 293.15 K and *p* = 100 000 Pa; each measurement is corrected using the air temperature and pressure measured at the time.

b The uncertainties in aperture transmission *k*_1_ and wall transmission *k*_p_ are negligible.

**Table 5:** Correction factors used in the comparison for the Ritz standard

Correction factor	Generating potential (kV)						Relative standard uncertainty (%)	
	30	25	50(b)	50(a)	80	100	Type A	Type B
Air attenuation *k*_a_^a^	1.054 3	1.039 5	1.011 7	1.005 9	1.005 4	1.004 5	0.03	0.01
Scattered radiation *k*_sc_	0.994 1	0.994 4	0.995 6	0.996 3	0.996 6	0.996 8		0.20
Electron loss *k*_e_	1.000 0	1.000 0	1.000 0	1.000 0	1.001 2	1.007 4		0.10
Ion recombination *k*_s_	1.000 4	1.000 1	1.000 1	1.000 0	1.000 0	1.000 2	0.04	
Field distortion *k*_d_	1.000	1.000	1.000	1.000	1.000	1.000		0.20
Aperture transmission *k*_1_	1.000 0	1.000 0	1.000 0	1.000 0	1.000 0	1.000 0		0.04
Wall transmission *k*_p_	1.000 0	1.000 0	1.000 0	1.000 0	1.000 0	1.000 0		0.01
Humidity *k*_h_	0.998	0.998	0.998	0.998	0.998	0.998		0.10

a These are nominal values for *T* = 293.15 K and *p* = 100 000 Pa; each measurement is corrected using the air temperature and pressure measured at the time.

**Table 6:** Correction factors used in the comparison for the BIPM standard

Correction factor	Generating potential (kV)							Relative standard uncertainty (%)	
	10	30	25	50(b)	50(a)	80	100	Type A	Type B
Air attenuation *k*_a_^a^	1.192 1	1.042 4	1.030	1.009 1	1.004 6	1.004 2	1.003 5	0.03	0.01
Scattered radiation *k*_sc_	0.994 4	0.995 6	0.995 7	0.996 6	0.997 1	0.997 4	0.997 4		0.07
Electron loss *k*_e_	1.000 0	1.000 0	1.000 0	1.000 0	1.000 0	1.010 0	1.020 7		0.01^b^
Ion recombination *k*_s_^c^	1.000 5	1.000 7	1.000 5	1.000 6	1.000 5			0.02	0.01
Ion recombination *k*_s_^d^	1.000 7	1.001 9	1.001 0	1.001 1	1.000 6	1.000 6	1.000 8	0.02	0.01
Field distortion *k*_d_	1.000 0	1.000 0	1.000 0	1.000 0	1.000 0	1.000 0	1.000 0		0.07
Aperture transmission *k*_1_	1.000 0	1.000 0	1.000 0	1.000 0	1.000 0	0.999 7	0.999 7		0.01
Wall transmission *k*_p_	1.000 0	1.000 0	1.000 0	1.000 0	1.000 0	1.000 0	1.000 0	0.01	
Humidity *k*_h_	0.998	0.998	0.998	0.998	0.998	0.998	0.998		0.03

a These are nominal values for *T* = 293.15 K and *p* = 100 000 Pa; each measurement is corrected using the air temperature and pressure measured at the time.

b For qualities above 50 kV, the value is 0.15 %.

c For an aperture of diameter 4.9992 mm.

d For an aperture of diameter 9.941 mm.

**Table 7:** Relative standard uncertainties in % associated with the comparison results

Source of uncertainty	NIST Lamperti		NIST Ritz BIPM			
	Type A	Type B	Type A	Type B	Type A	Type B
Ionization current	0.05	0.02	0.05	0.02	0.05	0.02
Volume	0.04	0.01	0.04	0.01	0.03	0.05
Positioning	0.01	0.03	0.01	0.03	0.01	0.01
Correction factors (excl. *k*_h_)	0.05	0.30	0.05	0.30	0.04	0.10^a^
Humidity *k*_h_		0.10		0.10		0.03
Physical constants		0.15		0.15		0.15

Quadratic sum	0.08	0.35	0.08	0.35	0.07	0.19^a^
Standard uncertainty of ${\dot{K}}_{\text{LAB}}$	0.36		0.36		0.20^a^	
Standard uncertainty of ${\dot{K}}_{\text{NIST}}/{\dot{K}}_{\text{BIPM}}$	0.34		0.34^b^			

a Applies up to 50 kV only. The increase in the uncertainty of *k*_e_ for the BIPM standard from 0.01 % to 0.15 % at 80 kV and 100 kV increases the uncertainty of ${\dot{K}}_{\text{BIPM}}$ to 0.25 % for these qualities.

b The increased uncertainty of *k*_e_ for the BIPM standard at 80 kV and 100 kV increases the uncertainty of the ratio ${\dot{K}}_{\text{RITZ}}/{\dot{K}}_{\text{BIPM}}$ to 0.37 % for these qualities.

**Table 8:** Results of the comparison of the NIST Lamperti standard with the BIPM standard

Generating potential (kV)	Aluminium half-value layer (mm)	${\dot{K}}_{\text{NIST}}/{\dot{K}}_{\text{BIPM}}$ 1966	${\dot{K}}_{\text{NIST}}/{\dot{K}}_{\text{BIPM}}$ 1998
10	0.036	0.9976	0.9950
30	0.176	0.9989	0.9961
25	0.250		0.9968
50(b)	1.021	0.9966	0.9948
50(a)	2.257	0.9948	0.9938

**Table 9:** Results of the comparison of the NIST Ritz standard with the BIPM standard

Generating potential (kV)	Aluminium half-value layer (mm)	${\dot{K}}_{\text{NIST}}/{\dot{K}}_{\text{BIPM}}$
30	0.176	0.9943
25	0.250	0.9949
50(b)	1.021	0.9938
50(a)	2.257	0.9956
80	3.01	0.9902
100	4.00	0.9947
